# Linking Mechanisms in the Intergenerational Transmission of Mental Health: The Role of Sex in Parent–Adolescent Dynamics

**DOI:** 10.3390/children11121484

**Published:** 2024-12-05

**Authors:** Hye-Jung Yun, Jungyeong Heo, Cynthia B. Wilson

**Affiliations:** 1 The Florida Center for Prevention Research, Florida State University, Tallahassee, FL 32301, USA; cbwilson2@fsu.edu; 2 Counseling & Psychological Services, Purdue University, West Lafayette, IN 47907, USA; heo42@purdue.edu

**Keywords:** adverse childhood experiences (ACEs), family resilience, ability to flourish, mental health

## Abstract

Background/Objectives: This study addresses the significance of mental health concerns by examining the intergenerational transmission of mental health between parents and adolescents. It investigates the serial mediating effects of family resilience, adolescents’ adverse childhood experiences (ACEs), and their ability to flourish in the transmission of mental health from parents to adolescents, with a focus on sex differences. Methods: This study used a sample of 54,434 adolescents aged 12–17 from the 2016–2020 National Survey of Children’s Health (NSCH). Mothers reported on their mental health status and family resilience, and adolescents’ ACEs, flourishing, and mental health problems including depression and anxiety. Serial mediation models were used to assess the hypotheses. Results: The findings revealed that better parental mental health status was associated with fewer mental health problems in adolescents, with significant sex differences observed in these associations. Specifically, in both maternal and paternal models, better parental mental health was linked to higher family resilience, which was associated with fewer ACEs, greater flourishing, and ultimately fewer adolescent mental health problems. Furthermore, maternal mental health had a stronger association with daughters’ mental health, while paternal mental health more strongly influenced sons’. Conclusions: These results highlight the importance of targeted prevention and clinical interventions to disrupt the intergenerational transmission of mental health issues.

## 1. Introduction

The intergenerational transmission of mental health has garnered increasing attention from researchers over the past decade [1]. According to the National Alliance on Mental Illness [2], 20% of adults and 17% of adolescents aged 12–17 in the United States have a mental illness, and globally, 15–23% of children live with a parent who has a mental health diagnosis. The link between the mental health of parents and their children is well established, with poor parental mental health being associated with an increased risk of mental health issues in children’s mental health, including anxiety and depression [3,4,5]. Given these factors, the intergenerational transmission of mental health concerns is particularly important during adolescence, a developmental period marked by significant emotional and cognitive changes.

Adverse childhood experiences (ACEs) encompass a wide range of traumatic and stressful events that can negatively impact children’s lives. While much of the research on ACEs has focused on children and adults, there is a growing need to explore adolescence, a developmental stage marked by rapid physical, cognitive, social, and emotional changes [6]. Adolescents exposed to ACEs face heightened mental health challenges, as traumatic experiences can disrupt emotional and cognitive development and are often compounded by a lack of positive family support [7]. Although studies have consistently shown a strong link between ACEs and mental health issues [8], there is limited understanding of the effect of ACEs on the intergenerational transmission of mental health between parents and adolescents. Thus, this study examined the mediating role of adolescents’ ACEs in the intergenerational transmission of mental health.

In contrast to research demonstrating the negative effects of ACEs, some studies have identified the protective roles of family and adolescent resilience in mitigating mental health issues [9,10]. Given the potential of resilience as a prevention or intervention tool, it is important to explore its role in the intergenerational transmission of mental health. Thus, this study investigated how family resilience and adolescents’ ability to flourish mediate the associations between parental mental health, adolescents’ ACEs, and their mental health outcomes.

### 1.1. Intergenerational Transmission of Mental Health

Although mental health symptoms are generally characterized by their impact on an individual’s functioning [11], substantial research highlights the relational nature of mental health issues. Family systems theory [12] offers a useful framework for understanding the intergenerational transmission of mental health, emphasizing that symptoms are often shaped by dynamic interactions within family systems. Research demonstrated that children of parents with poor mental health are more likely to experience mental health problems themselves [3]. A systemic lens emphasizes the interrelatedness of parents’ and adolescents’ mental health, suggesting that “symptoms represent recursive feedback cycles of escalated behavior and experience that are organized into an interactional system” (p. 124) [13]. For example, Reid [14] found that children living with a parent with poor mental health are more likely to have mood or anxiety disorders compared to those living with a parent without mental health symptoms. Similarly, Lui et al. [15] found that parental mental health directly impacted their children’s emotional outcomes. The systemic concept of nonsummativity suggests that “the behavior of an element is different within the system from what it is in isolation” (p. 58) [16]. Building on this, Zhou et al. [17] differentiate between general mental health challenges, which broadly impact a range of psychological and social outcomes, and specific issues, such as internalizing problems (e.g., depression, anxiety) and externalizing problems (e.g., behavioral issues), which are more closely linked to similar outcomes in children, including depression, anxiety, and conduct disorders. Therefore, a comprehensive understanding of the transmission of mental health should also consider additional factors, such as risk and protective factors, within the parent–adolescent subsystem.

### 1.2. Linking Mechanisms for Transmission of Mental Health Between Parents and Adolescents

While parental mental health plays a significant role in their children’s mental health, external risk factors, such as adverse childhood experiences (ACEs), can exacerbate mental health challenges for adolescents. On the other hand, protective factors, like family resilience and adolescents’ ability to flourish may help buffer the negative impacts of these experiences. Exploring the interaction between these factors offers a deeper understanding of how mental health is transmitted across generations.

Adolescents’ ACEs: ACEs are defined as traumatic experiences during childhood that can impact individuals’ health and functioning throughout their lifetime [18,19]. These experiences include parental divorce/separation, the death of a parent or guardian, domestic violence, substance abuse, mental illness, financial hardship, exposure to violence, discrimination, and the incarceration of a family member before the age of 18 [20].

Parental mental illness, a specific type of ACE, can directly impact adolescents’ mental health. Further, ACEs can act as a pathway linking parental mental health to adolescents’ mental health outcomes. For example, when parents suffer from mental illness, it can lead to an increase in ACEs for their children. In turn, adolescents exposed to ACEs are more likely to exhibit poor mental health outcomes [21,22]. That is, ACEs can lead to a decline in adolescents’ mental health, putting them at a higher risk for psychological disorders such as anxiety and depression. Thus, this study examined the mediating effect of adolescents’ ACEs on the relationship between parental mental health status and adolescents’ mental health problems.

Family Resilience: Given the negative health outcomes associated with ACEs, it is important to identify protective factors for adolescents who have experienced adversities. In resilience research, the terms “promotive” and “protective” factors have been used to describe variables that contribute to adaptive outcomes under conditions of adversity. Promotive factors are associated with positive outcomes regardless of the level of risk, while protective factors specifically mitigate the adverse effects of high-risk environments [23]. In this study, we adopt the term “protective factors” to emphasize their buffering role in reducing the negative impact of adversity, while acknowledging that these factors may also possess promotive qualities in broader contexts. A growing body of research suggests that high levels of family resilience can serve as a protective factor in the face of adverse experiences [9,24]. Family resilience refers to the ability of the family to adapt to change and overcome significant stressors [25]. Factors associated with family resilience include frequent family communication, effective problem-solving, family strength, and positivity. Recent efforts in clinical practices advocate that family resilience has the potential to mitigate the adverse effects of ACEs on adolescents’ mental health. For example, Uddin et al. [9] found that family resilience could reduce the impact of ACEs on children’s mental health. However, it remains unclear how family resilience mediates the association among parental mental health status, adolescents’ ACEs, and their mental health problems. Therefore, this study examined the mediating role of family resilience in such associations.

Adolescents’ Ability to Flourish: Adolescents’ ability to flourish is recognized as a foundational component of positive well-being and as a protective factor against mental health problems [26,27]. In 2011/12, the National Survey of Children’s Health (NSCH) introduced indicators of flourishing to provide positive measures of health, as recommended by a Technical Expert Panel (TEP). This concept of flourishing in adolescents includes curiosity in learning, resilience, and self-regulation. Bethell et al. [10] found that adolescents exposed to ACEs who displayed demonstrated resilience were significantly less likely to experience emotional, mental, and behavioral problems compared to those with lower resilience. This suggests that the ability to flourish could act as a possible mediator between adolescents’ ACEs and mental health outcomes. Therefore, this study examined the mediating effect of adolescents’ ability to flourish in the relationship between parental mental health status, adolescents’ ACEs, and their mental health problems.

### 1.3. Sex-Specific Pathways

Previous research indicated that parental mental health may affect sons and daughters differently during adolescence. Connell and Goodman’s meta-analysis [28] found that while maternal mental health generally had a stronger association with children’s outcomes than paternal mental health, the differences were relatively small, suggesting the importance of both parents’ mental health. Specifically, Goodman et al. [29] found that maternal mental health problems have a stronger impact on daughters compared to sons, potentially due to sex-specific modeling and identification processes [30]. These sex-specific pathways highlight the importance of considering child sex within broader family dynamics, such as family resilience, adolescents’ ACEs, and their ability to flourish, to fully understand how parental mental health influences adolescent outcomes. Thus, this study examined the sex differences within our research model.

### 1.4. The Present Study

**Figure 1:**
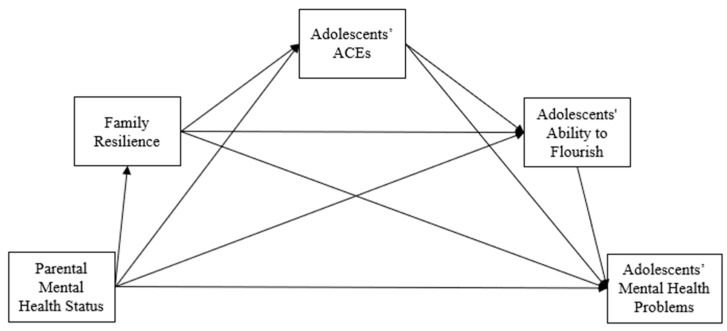
The proposed serial mediation model.

Given the importance of intergenerational transmission of mental health, this study aims to address existing gaps in the literature by examining how family resilience, adolescents’ ACEs, and their ability to flourish mediate the relationship between parental and adolescent mental health. Based on previous theoretical and empirical research, we proposed three hypotheses: First, based on family systems theory [12], parental mental health status would be negatively associated with adolescents’ mental health problems (H1). Second, the relationship between parental mental health status and adolescent mental health problems would be serially mediated by family resilience, adolescents’ ACEs, and their ability to flourish (H2), as shown in Figure 1. Lastly, based on sex-specific modeling [30], the strength of these relationships would vary by parent and adolescent sex. Specifically, we hypothesized that maternal mental health would have a stronger association with daughters’ mental health problems than sons’, while paternal mental health would have a stronger association with sons’ mental health problems than daughters’ (H3).

## 2. Methods

### 2.1. Samples and Procedures

Data for this study were derived from the 2016–2020 National Survey of Children’s Health (NSCH), a nationally representative, cross-sectional survey conducted annually by the U.S. Census Bureau, sponsored by the U.S. Maternal and Child Health Bureau, and maintained by the Child and Adolescent Health Measurement Initiative (CAHMI) [31]. The NSCH assesses various aspects of child and family health-related experiences across the United States. Following the NSCH Guide to Multi-Year Analysis [32], we merged data from five consecutive years (2016–2020) to increase the sample size and enhance the representativeness of the findings. Sampling weights from the NSCH public-use dataset were applied to account for non-response and selection probabilities, ensuring results could be generalized to the U.S. parent and adolescent population. The final sample size was 54,434 parents with adolescents aged 12–17.

### 2.2. Measures

#### 2.2.1. Parental Mental Health Status

Paternal and maternal mental health were each assessed with a single item, where each parent reported their mental health status. Responses were rated on a three-point Likert scale, recoded as 1 = fair/poor, 2 = good, and 3 = excellent/very, so that higher scores indicated better overall paternal or maternal mental health.

#### 2.2.2. Adolescents’ Mental Health Problems

Parents reported on their adolescent children’s (ages 12–17) depression and anxiety symptoms. These two items were combined to create an overall indicator of adolescents’ mental health problems. Responses were coded as 0 = does not have a condition and 1 = does have a condition. The items of anxiety and depression were summed, with higher scores indicating greater levels of mental health problems in adolescents.

#### 2.2.3. Adolescents’ ACEs

Parents reported their adolescent children’s ACEs through nine items assessing exposures to (1) financial hardship, (2) parent/guardian separation or divorce, (3) death of parent/guardian, (4) parent/guardian incarceration, (5) domestic violence, (6) victim/witness of neighborhood violence, (7) mental health issues, (8) alcohol/drug abuse, and (9) race/ethnicity discrimination. All items were recoded as 0 = no and 1 = yes, and the items were summed to create the ACE scores, with higher scores indicating a greater number of ACEs. Previous studies have demonstrated good internal consistency reliability (e.g., α = 0.86) [33] and validity of ACEs scales, showing a significant association with adolescent depression (β = 0.29, *p* < 0.001) and anxiety (β = 0.26, *p* < 0.001) in a variety of samples [34]. McDonald’s omega for this measure was 0.66.

#### 2.2.4. Family Resilience

Family resilience was measured using four items that captured qualities of how families respond to difficulties. Participants rated how often their family engages in the following behaviors: (1) talk together about what to do; (2) work together to solve our problems; (3) know we have strengths to draw on; and (4) stay hopeful even in difficult times. Each item was scored on a three-point Likert scale, recoded as 1 = some/none of the time, 2 = most of the time, and 3 = all of the time. Items were summed to create a total family resilience score, with higher scores indicating greater family resilience. Previous studies have demonstrated strong internal reliability (e.g., α = 0.92) [35] and construct validity, showing a significant association with adolescent depression (β = −0.71, *p* < 0.001) [36]. In the current sample, McDonald’s omega was 0.89.

#### 2.2.5. Adolescents’ Ability to Flourish

Three questions were used to assess adolescents’ curiosity about learning, resilience, and self-regulation for adolescents aged 12–17. These questions asked parents if their adolescent children (1) show interest and curiosity in learning new things, (2) work to finish tasks he or she start, and (3) stay calm and in control when faced with a challenge. Each item was recoded as 0 = no and 1 = yes, then summed to create an adolescent’s flourishing scores. A higher score reflected a higher level of an adolescent’s ability to flourish. Earlier studies have demonstrated good internal reliability (e.g., α = 0.75) [37] and validity, showing a significant association with family resilience (β = 0.29, *p* < 0.001) [38]. McDonald’s omega for this measure was 0.73.

#### 2.2.6. Adolescent Sex

Adolescent sex (coded as 1 = female, 0 = male) was added as a moderator to investigate potential sex differences in the research model, given evidence that sex may influence the intergenerational transmission of mental health. Previous research suggests that maternal and paternal mental health may impact sons and daughters differently [28].

### 2.3. Analytical Plan

We conducted path analyses using Mplus 8 [38] to test our hypotheses. The analysis proceeded in three stages.

First, we evaluated the direct effect of parental mental health status on adolescents’ mental health problems, expecting a significant path coefficient. This would suggest that better parental mental health is associated with fewer mental health problems in adolescents (H1). Second, we tested the serial mediation model, investigating whether the relationship between parental mental health status (maternal and paternal mental health status, separately) and adolescent mental health problems was mediated through three sequential pathways: family resilience, adolescents’ ACEs, and ability to flourish (H2). Each indirect path coefficient was examined to confirm the role of these mediating factors. Lastly, to test sex differences (H3), we conducted multi-group path analyses comparing the path coefficients across sex groups, allowing us to determine whether the strength or direction of associations varied by sex. To achieve this, we conducted a χ^2^ difference test between an unrestricted model, in which all path coefficients were freely estimated for each sex, and 10 separate restricted models, each constraining a single path coefficient to be equal across sexes. A significant χ^2^ difference between the unrestricted and restricted models indicates that the constrained path coefficient significantly differs by sex.

The significance of indirect effects was evaluated using bootstrap confidence intervals based on 5000 resamples. Cases with missing data on parental mental health status (independent variable) were excluded from analyses while missing data on other variables were handled using Full Information Maximum Likelihood (FIML). Model fits were assessed using chi-square ($\chi^{2})$, the root mean square error (RMSEA), the comparative index (CFI), and the Tucker–Lewis index (TLI). Models with an RMSEA below 0.05, along with CFI and TLI above 0.95, are considered to have great model fits [39].

## 3. Results

### 3.1. Preliminary Results

Descriptive statistics and correlations among the variables are shown in Table 1. From the correlations, both maternal and paternal mental health status was significantly and negatively correlated with adolescents’ mental health (*r* = −0.21 for maternal and *r* = −0.16 for paternal, *p* < 0.01), providing preliminary support for H1. Additionally, both maternal and paternal mental health status was significantly and negatively associated with adolescents’ ACEs (*r* = −0.31 for maternal and *r* = −0.27 for paternal, *p* < 0.01). Parental mental health was also significantly and positively associated with family resilience (*r* = 0.27 for maternal and *r* = 0.26 for paternal, *p* < 0.01) and adolescents’ ability to flourish (*r* = 0.15 for maternal and *r* = 0.11 for paternal; *p* < 0.01). Furthermore, adolescents’ ACEs were negatively associated with both family resilience (*r* = −0.16, *p* < 0.01) and adolescent flourishing (*r* = −0.16, *p* < 0.01), while family resilience was positively correlated with adolescents’ ability to flourish (*r* = 0.21, *p* < 0.01). These correlations offered preliminary support for H2. Adolescent sex (female =1) is associated with adolescents’ mental health problems (*r* = 0.08, *p* < 0.01) and adolescents’ ability (*r* = 0.05, *p* < 0.01). Based on these preliminary findings, we proceeded to hypothesis testing.

### 3.2. Hypothesis Testing for the Association Between Parental Mental Health Status and Adolescents’ Mental Health Problems (H1)

Path analysis results indicated that maternal/paternal mental health statuses were significantly and negatively associated with adolescents’ mental health problems (*β* = −0.20, *p* < 0.01 for maternal; *β* = −0.16, *p* < 0.01 for paternal), after controlling for other influential variables including adolescent sex. In other words, better maternal and paternal mental health were associated with fewer adolescent mental health problems.

### 3.3. Hypothesis Testing for the Mediating Effects of Family Resilience, Adolescents’ ACEs, and Their Ability to Flourish in the Intergenerational Transmission of Mental Health (H2)

**Figure 2:**
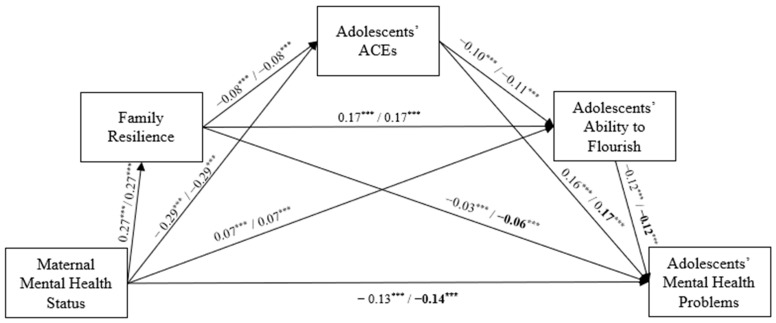
The serial mediation model from maternal mental health status to adolescents’ mental health problems. Note: The path coefficients are shown separately for adolescent sex (boys/girls). Bold indicates a significantly higher coefficient compared to the other sex. *** *p* < 0.001.

The serial mediation model for maternal mental health demonstrated a good fit ($\chi^{2}\left(3\right)=142.22,RMSEA=0.03\;\left[95\%\;CI\left(0.03,\;0.04\right)\right],\;CFI=0.99,\;TLI=0.96$). In the model, all regression paths were significant (see Figure 2). Specifically, maternal mental health status positively influenced familial resilience (*β* = 0.27, *p* < 0.001) and adolescents’ ability to flourish (*β* = 0.07, *p* < 0.001), while reducing adolescents’ ACEs (*β* = −0.29, *p* < 0.001) and mental health problems (*β* = −0.13, *p* < 0.001). Family resilience was associated with fewer adolescents’ ACEs (*β* = −0.08, *p* < 0.001) and mental health problems (*β* = −0.04, *p* < 0.001), and it increased adolescents’ ability to flourish (*β* = 0.17, *p* < 0.001). Additionally, adolescents’ ACEs were linked to a decrease in their ability to flourish (*β* = −0.11, *p* < 0.001) but an increase in mental health problems (*β* = 0.16, *p* < 0.001). Adolescents’ ability to flourish was inversely related to their mental health problems (*β* = −0.12, *p* < 0.001). As shown in Table 2, the bootstrapping results revealed significant indirect effects from maternal mental health status to adolescents’ mental health problems. These findings indicate that family resilience, adolescents’ ACEs, and their ability to flourish sequentially mediated the relationship between maternal mental health and adolescents’ mental health problems (*β* = 0.00, *z* = −12.17, *p* < 0.001).

**Figure 3:**
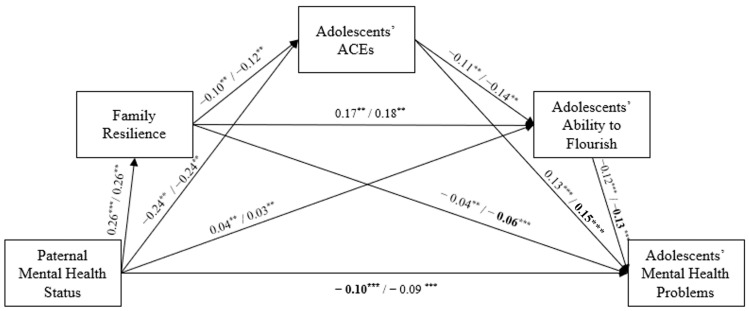
The serial mediation model from paternal mental health status to adolescents’ mental health problems. Note: The path coefficients are shown separately for adolescent sex (boys/girls). Bold indicates a significantly higher coefficient compared to the other sex. ** *p* < 0.01, *** *p* < 0.001.

The serial mediation model for paternal mental health demonstrated a good model fit ($\chi^{2}(3)=100.05,RMSEA=0.03\;\left[95\%\;CI\left(0.023,\;0.032\right)\right],\;CFI=0.99,\;TLI=0.96$). The regression coefficients in the serial mediation model of paternal mental health status mirrored those in the maternal model, with all regression paths showing significant effects (see Figure 3). Specifically, paternal mental health status positively influenced family resilience (*β* = 0.26, *p* < 0.001) and adolescents’ ability to flourish (*β* = 0.04, *p* < 0.001) while reducing adolescents’ ACEs (*β* = −0.24, *p* < 0.001) and mental health problems (*β* = −0.09, *p* < 0.001). Family resilience was associated with lower levels of adolescents’ ACEs (*β* = −0.11, *p* < 0.001) and mental health problems (*β* = −0.05, *p* < 0.001) while increasing adolescents’ ability to flourish (*β* = 0.17, *p* < 0.001). Adolescents’ ACEs reduced their ability to flourish (*β* = −0.12, *p* < 0.001) and were linked to increased mental health problems (*β* = 0.14, *p* < 0.001). Additionally, adolescents’ ability to flourish was negatively associated with mental health problems (*β* = −0.12, *p* < 0.001). As shown in Table 3, the bootstrapping results revealed significant indirect effects from paternal mental health status to adolescents’ mental health problems. These findings show that family resilience, adolescents’ ACEs, and their ability to flourish sequentially mediated the association between paternal mental health status and adolescents’ mental health problems (*β* = 0.00, *z* = −13.36, *p* < 0.001).

### 3.4. Hypothesis Testing for Sex Difference in the Serial Mediation Model (H3)

Some regression coefficients revealed sex differences. In the maternal mental health model, maternal mental health (${\Delta \chi }^{2}$ *=* 47.88)*,* family resilience (${\Delta \chi }^{2}$ *=* 47.28), adolescents’ ACEs (${\Delta \chi }^{2}$ *=* 49.77), and their ability to flourish (${\Delta \chi }^{2}$ *=* 47.51) similarly impact adolescent mental health problems, with stronger effects observed among girls (maternal mental health status: *β* = −0.14; family resilience: *β* = −0.06; adolescents’ ACEs: *β* = 0.17; and adolescents’ ability to flourish: *β* = −0.12) than boys (maternal mental health status: *β* = −0.13; family resilience: *β* = −0.03; adolescents’ ACEs: *β* = 0.16; and adolescents’ ability to flourish: *β* = −0.12).

In the paternal mental health model, family resilience (${\Delta \chi }^{2}$ *=* 35.73), adolescents’ ACEs (${\Delta \chi }^{2}$ *=* 52.11), and adolescents’ ability to flourish (${\Delta \chi }^{2}$ *=* 37.84) also have similar impacts on adolescents’ mental health problems across sex. However, paternal mental health (${\Delta \chi }^{2}$ = 21.39) has a slightly stronger impact on boys’ mental health problems (*β* = −0.10) than on girls (*β* = −0.09)

## 4. Discussion

Given the public health importance of mental health, this study examined the intergenerational transmission of mental health, focusing on the mediating roles of family resilience, adolescents’ ACEs, and their ability to flourish in the association between parental mental health and adolescents’ mental health problems. Ground in family systems theory [11], we hypothesized that parental mental health status would directly influence adolescent mental health (H1). Furthermore, we proposed that family resilience, adolescents’ ACEs, and their ability to flourish would mediate the intergenerational transmission of mental health between parents and adolescents (H2). Additionally, we explored sex differences in these pathways (H3), hypothesizing that maternal mental health would have a stronger impact on daughters’ mental health, while paternal mental health would more strongly affect sons’.

### 4.1. The Impact of Parental Mental Health on Adolescent Mental Health (H1)

Consistent with our hypothesis and previous research [3,4,5], we found that both maternal and paternal mental health were significantly and negatively associated with adolescents’ mental health problems. This finding underscores the critical influence of parental mental health on adolescent well-being, aligning with family systems theory [12], which emphasizes the interconnectedness of family members’ mental health. The relational nature of mental health within families suggests that improving parental mental health may have a beneficial impact on adolescents, potentially reducing the risk of developing mental health issues.

### 4.2. Mediating Effects of Family Resilience, Adolescents’ ACEs, and Adolescents’ Ability to Flourish (H2)

Our findings support the second hypothesis, showing that family resilience, adolescents’ ACEs, and their ability to flourish serve as serial mediators in the relationship between parental and adolescent mental health. In both maternal and paternal models, better parental mental health was associated with higher family resilience, which in turn was related to fewer ACEs and greater flourishing in adolescents, ultimately resulting in fewer adolescent mental health problems.

As a risk factor, the significant mediating role of ACEs in the relationship between parental mental health and adolescent outcomes aligns with previous research on how adverse experiences impact mental health [21,22]. The negative association between parental mental health and adolescents’ ACEs suggests that better parental mental health may serve as a protective factor against adverse experiences, possibly through more effective parenting practices or resilient home environments [9,24].

These findings further support the notion that family resilience can serve as a protective factor [9,24], helping to mitigate the adverse effects of ACEs on adolescent mental health. Defined as a family’s capacity to adapt to stress and work together to overcome challenges [25], family resilience is essential for buffering adolescents against the negative impacts of ACEs. This resilience likely creates an environment that promotes positive coping strategies and emotional support, reducing the risk of adolescents developing mental health problems in the face of adversities.

Additionally, the adolescents’ ability to flourish, which encompasses curiosity, resilience, and self-regulation, emerged as a significant mediator, supporting previous research on its protective role in mental health outcomes [26,27]. This finding highlights the importance of fostering positive well-being indicators in adolescents as a protective factor against mental health issues. Adolescents who exhibit greater resilience and self-regulation may be better equipped to manage stress, reducing the likelihood of experiencing mental health problems. Together, these mediating factors provide insight into specific protective mechanisms that could help interrupt the transmission of mental health problems across generations.

### 4.3. Sex-Specific Pathways in the Intergenerational Transmission of Mental Health (H3)

Our findings also support the hypothesis that the pathways linking parental mental health to adolescent mental health outcomes vary by sex. In the maternal health model, the effects of maternal mental health, family resilience, adolescents’ ACEs, and their flourishing on adolescent mental health problems were stronger for girls than for boys. This is consistent with research suggesting that maternal mental health often has a more pronounced impact on daughters, potentially due to closer emotional bonds, sex-specific socialization, or identification processes [30]. However, the differences were relatively modest, suggesting that maternal mental health significantly influences both sons and daughters and that improving maternal mental health could have a more substantial protective effect on their adolescent children’s well-being.

In the paternal mental health model, family resilience, adolescents’ ACEs, and their flourishing had similar impacts on adolescent mental health for both sexes. However, paternal mental health had a slightly stronger effect on boys’ mental health problems compared to girls’. This aligns with previous research suggesting that sons may be more influenced by their fathers’ mental health, possibly due to sex-specific identification processes [29]. These findings underscore the importance of considering both parental and adolescent sex in understanding the transmission of mental health within families.

### 4.4. Limitations and Future Directions

Despite its contributions and strengths, this study has limitations primarily due to the use of secondary data. First, the cross-sectional nature of the data limits causal inferences about the observed relationships. Future studies could consider longitudinal designs to better capture the temporal sequences of intergenerational transmission of mental health, e.g., [40,41]. Specifically, previous longitudinal studies provided contrasting insights into cross-lagged effects between parent and child mental health. Griffith et al. [42] found contemporaneous cofluctuation of depressive symptoms but no evidence of bidirectional effects over time. In contrast, Yirmiya et al. [43] identified bidirectional influences between maternal and child anxiety, particularly in trauma-exposed families, with early childhood as a sensitive period. These findings highlight the complexity of intergenerational mental health transmission and the importance of contextual factors like trauma and developmental stages. Future longitudinal studies would benefit from incorporating complex contextual factors when examining intergenerational transmission of mental health. Second, the reliance on parent-reported measures may introduce reporting bias, particularly for adolescent outcomes. Future research could improve accuracy by incorporating multiple informants, objective measures, and adolescent self-reports [44,45]. Third, each maternal and paternal mental health status was accessed using single measures, which may not fully capture the complexity of mental health status. Future studies could benefit from more comprehensive assessments of parental mental health, as well as additional contextual factors that may influence family dynamics. Fourth, while this study focused on examining sex differences within the serial mediation model, we did not include covariates. Future studies could consider sociodemographic factors such as adolescent age, race/ethnicity, educational background, and income, which may further influence the relationships between parental mental health, family resilience, adolescents’ ACEs, and their flourishing. Lastly, while this study focuses on adolescents, future research could explore how sex-specific pathways operate across different developmental stages for a more comprehensive understanding.

### 4.5. Conclusions and Implications

Using a large nationally representative sample from the combined 2016–2020 NSCH data, this study provides significant contributions to understanding the intergenerational transmission of mental health by examining the mediating roles of family resilience, adolescents’ ACEs, and their flourishing in the association between parental and adolescent mental health. By identifying key mediating mechanisms and exploring sex-specific pathways, this study enhances our understanding of the complex dynamics through which parental mental health influences adolescent mental health outcomes. The findings emphasize the importance of addressing parental mental health, enhancing family resilience, and fostering adolescents’ ability to flourish as protective strategies to mitigate mental health risks across generations. Specifically, the role of family resilience suggests that interventions focusing on family level protective factors could be effective in preventing the transmission of mental health problems. Programs that enhance family communication, problem-solving skills, and collective coping strategies may be particularly impactful, helping families manage stress and reducing adolescent susceptibility to mental health challenges in the context of ACEs.

These findings carry practical implications for policymakers and practitioners seeking to reduce the intergenerational transmission of mental health issues. First, policies and programs that support parental mental health can foster a more resilient family environment, reducing children’s exposure to adverse experiences and promoting family dynamics. Access to mental health resources for parents could be prioritized to interrupt the type of mental health challenges across generations. Second, clinicians and practitioners are encouraged to implement family centered prevention strategies that enhance resilience at the family level. Also, programs aimed at fostering positive well-being indicators in adolescents, such as curiosity, resilience, and self-regulation, are vital for mental health prevention. Schools, community organizations, and mental health services can support adolescents in developing these attributes, which may enhance their capacity to handle stress and reduce the risk of mental health issues. Lastly, the observed sex differences suggest that tailored interventions may be necessary to address the distinct needs of sons and daughters. For example, programs that support maternal mental health may focus on enhancing mother–daughter interactions, while those targeting paternal mental health may prioritize father–son dynamics, recognizing the unique influences of each parental role on adolescent mental health.

In conclusion, this study offers important insights into the mechanisms of intergenerational mental health transmission and highlights the protective roles of family resilience and adolescents’ flourishing. Targeted policies and interventions that enhance family resilience, support parental mental health, and foster adolescents’ well-being may be critical in reducing the transmission of mental health issues across generations. These findings provide a foundation for developing evidence-based strategies aimed at promoting family resilience and mental health stability across generations.

## Figures and Tables

**Table 1:** Descriptive statistics and correlations among study variables.

Variables	M or % (SD)	1	2	3	4	5	6
1. Maternal/paternal mental health status	M: 2.71 (0.55) P: 2.76 (0.51)	_	−0.16 **	−0.27 **	0.26 **	0.11 **	0.00
2. Adolescents’ mental health problems	0.21 (0.53)	−0.21 **	_	0.23 **	−0.13 **	−0.17 **	0.08 **
3. Adolescents’ ACEs	1.34 (1.48)	−0.31 **	0.23 **	_	−0.16 **	−0.16 **	0.01
4. Family resilience	9.22 (2.35)	0.27 **	−0.13 **	−0.16 **	_	0.21 **	0.00
5. Adolescents’ ability to flourish	2.43 (0.95)	0.15 **	−0.17 **	−0.16 **	0.21 **	_	0.05 **
6. Adolescent sex (1 = female)	48.5%	0.00	−0.08 **	0.01	0.00	0.05 **	_

Note: Correlations for the maternal sample are shown below the diagonal (N = 48,424); correlations for the paternal sample are shown above the diagonal (N = 42,279). ** *p* < 0.01; two-tailed tested.

**Table 2:** Indirect, direct, and total effects of maternal mental health status on adolescents’ mental health problems.

Model Pathway	Estimate [95% CI ^†^]	
	Boys	Girls
Maternal MH → ACEs → adolescent MHP	−0.045 *** (−0.049, −0.041)	−0.047 *** (−0.052, −0.043)
Maternal MH → flourish → adolescent MHP	−0.009 *** (−0.010, −0.007)	−0.008 *** (−0.010, −0.006)
Maternal MH → family resilience → adolescent MHP	−0.008 *** (−0.011, −0.004)	−0.015 *** (−0.019, −0.012)
Maternal MH → family resilience → ACES → adolescent MHP	−0.003 *** (−0.004, −0.003)	−0.004 *** (−0.004, −0.003)
Maternal MH → ACES → flourish → adolescent MHP	−0.003 *** (−0.004, −0.003)	−0.004 *** (−0.005, −0.003)
Maternal MH → family resilience → flourish → adolescent MHP	−0.005 *** (−0.006, −0.005)	−0.006 *** (−0.006, −0.005)
Maternal MH → family resilience → ACES → flourish → adolescent MHP	0.000 *** (0.000, 0.000)	0.000 *** (0.000, 0.000)
Total indirect effect: maternal MH → adolescent MHP	−0.074 *** (−0.079, −0.068)	−0.084 *** (−0.090, −0.079)
Direct effect: maternal MH → adolescent MHP	−0.131 *** (−0.144, −0.118)	−0.135 *** (−0.149, −0.122)
Total effect of maternal MH: total indirect effect + direct effect	−0.205 *** (−0.217, −0.193)	−0.220 *** (−0.232, −0.208)

Note: *** *p* < 0.001; two-tailed tested. ^†^ Symmetric confidence interval.

**Table 3:** Indirect, direct, and total effects of paternal mental health status on adolescents’ mental health problems.

Model Pathway	Estimate [95% CI ^†^]	
	Boys	Girls
Paternal MH → ACEs → adolescent MHP	−0.031 *** (−0.034, −0.027)	−0.036 *** (−0.040, −0.033)
Paternal MH → flourish → adolescent MHP	−0.005 *** (−0.007, −0.003)	−0.004 *** (−0.005, −0.002)
Paternal MH → family resilience → adolescent MHP	−0.009 *** (−0.013, −0.006)	−0.016 *** (−0.019, −0.012)
Paternal MH → family resilience → ACES → adolescent MHP	−0.003 *** (−0.004, −0.003)	−0.005 *** (−0.005, −0.004)
Paternal MH → ACES → flourish → adolescent MHP	−0.003 *** (−0.004, −0.003)	−0.004 *** (−0.005, −0.003)
Paternal MH → family resilience → flourish → adolescent MHP	−0.006 *** (−0.006, −0.005)	−0.006 *** (−0.007, −0.005)
Paternal MH → family resilience → ACES → flourish → adolescent MHP	0.000 *** (0.000, 0.000)	−0.001 *** (−0.001, 0.000)
Total indirect effect: paternal MH → adolescent MHP	−0.057 *** (−0.063, −0.052)	−0.071 *** (−0.076, −0.065)
Direct effect: paternal MH → adolescent MHP	−0.096 *** (−0.109, −0.082)	−0.135 *** (−0.034, −0.027)
Total effect of paternal MH: total indirect effect + direct effect	−0.153 *** (−0.166, −0.140)	−0.163 *** (−0.176, −0.149)

Note: *** *p* < 0.001; two-tailed tested. ^†^ Symmetric confidence interval.

## Data Availability

Data used in this study are publicly accessible through the National Survey of Children’s Health website at https://www.childhealthdata.org/help/dataset (accessed on 20 June 2022).
